# Spontaneous secondary pneumothorax due to cannabis-induced bullous lung disease: a case report

**DOI:** 10.1097/MS9.0000000000000968

**Published:** 2023-06-20

**Authors:** Vladislav Pavlovich Zhitny, Jared Diaz, Chalette Lambert-Swainston, Ala Abdallah, Sana Gurz, Jake Young, Chriselyn Palma, Cliff Chen, Nazia Khan

**Affiliations:** a Department of Anesthesiology, New York University, Perioperative Care and Pain Medicine, New York City, NY; b Department of Internal Medicine; c Department of Pulmonary, Critical Care Medicine; d University of Nevada; e Department of Biology, University of Utah, Salt Lake City, UT

**Keywords:** Spontaneous pneumothorax, marijuana smoking, cannabis abuse, tobacco smoking, lung bulla

## Abstract

Marijuana use has grown rapidly in the last decade with a prevalence greater than that of cocaine and opioids. With its increasing recreational and medical use, potential adverse outcomes from heavy use may be associated with bullous lung disease and spontaneous pneumothorax.

This case report has been reported in line with the SCARE Criteria.

**Case presentation:**

The authors describe a case of an adult male with a past medical history of spontaneous pneumothorax and long-standing marijuana use presenting with dyspnoea who was found to have a secondary spontaneous pneumothorax requiring invasive treatment.

**Clinical discussion:**

The aetiology of lung injury due to heavy marijuana smoke may be from direct tissue injury from inhaled irritants and the method of which marijuana smoke is inhaled compared with tobacco smoke.

**Conclusion:**

Chronic marijuana use should be considered when evaluating structural lung disease and pneumothorax in the setting of minimal tobacco use.

## Introduction

Highlights

- Chronic marijuana use should be considered when evaluating for etiologies of structural lung disease that leads to a secondary spontaneous pneumothorax.
- It is important to keep in mind that structural abnormalities from other organ systems can contribute to the development of a spontaneous pneumothorax.
- This case highlights the potential risks of marijuana abuse to lung health in the setting of minimal tobacco use.

The use of marijuana in the United States has risen rapidly in the past decade, especially with growing support both legislatively and medically^1^. Marijuana has been increasingly prescribed by providers in some conditions, including HIV/AIDs wasting syndrome, cancer and chemotherapy treatment, and pain^2^. However, the widespread use of marijuana, both recreationally and therapeutically, has raised the question of short term and long-term respiratory effects. Marijuana use has been associated with respiratory symptoms such as increased sputum production, wheezing, and cough, along with physiological changes such as bronchodilation. The technique in which marijuana is smoked, compared with tobacco inhalation, may also predispose the smokers to barotrauma^3–10^.

Other documented pulmonary consequences in habitual marijuana use are an increased risk of lung malignancy, pneumonia, and development of emphysema. Histopathological changes in the airspaces and respiratory tracts of marijuana smokers have been demonstrated in smaller studies. Additionally, fungal spores in cannabis plants may be inhaled and cause fungal pneumonia in some immunocompromised patients, a population in which marijuana may be commonly prescribed^9^. Finally, bullous emphysema development has been associated with marijuana smoking in patients presenting with spontaneous pneumothorax^3^–^9^,^11^.

We report an interesting case of a 46-year-old male with a past medical history significant for recurrent pneumothoraces, marijuana use disorder, and minimal tobacco use found to have a spontaneous non-tension right pneumothorax. Written informed consent was obtained from the patient for publication of this case report and accompanying images. A copy of the written consent is available for review by the Editor-in-Chief of this journal on request.

## Case presentation

A 46-year-old. Hispanic male with a past medical history significant for recurrent pneumothoraces, cannabis smoking, minimal tobacco smoking, heart failure with mildly reduced ejection fraction (45–50%), history of pericardial effusion, and cardiac tamponade status post-pericardial window presented to a major trauma with complaints of sudden onset chest pain and hemoptysis.

The patient had been experiencing fevers, chills, and worsening shortness of breath for the past 2 weeks. He had a remote history of prior left-sided tension pneumothorax in 2001 due to a stab wound that required a chest tube placement. Two years later, the patient had a right-sided spontaneous pneumothorax that required chest tube placement that was thought to be secondary to marijuana use. The patient has been smoking 7.5 g of marijuana per day since his early teenage years (for over 20 years). He also smoked half a cigarette per day for the past 6.75 years. He denied any history of asthma or Chronic Obstructive Pulmonary Disease and was not taking any medications at home with no history of any prescribed medication use. The patient also reported to have a history of a large ventral hernia. In 2018, the patient presented to another community hospital with acute onset shortness and abdominal pain. He was found to have a left diaphragmatic hernia (possibly secondary to previous trauma) for which he underwent operative repair. He had a complicated postoperative course to include midline wound dehiscence, for which pt reports a “skin substitute” was placed. He reported occasionally wearing an abdominal binder to keep the hernia in place; stated that he had “twinges” of right-sided pain when the binder was removed, but he was still passing gas and having regular bowel movements.

The patient’s symptoms began after bearing down in the restroom. He developed hemoptysis along with a sensation of an “air bubble” in his chest which felt similar to his prior pneumothoraces. The patient drove to the emergency department for evaluation. On admission, the patient reported right-sided, non-radiating chest pain that he rated a 10/10. The patient was afebrile at 36.4 °C with a heart rate of 74/min, blood pressure of 132/88 mmHg, respirations of 18 breaths/min, and SpO_2_ of 98% on room air. He had a BMI 21.03 kg/m^2^. Electrocardiogram showed normal sinus rhythm without any QTc prolongation. Physical examination was significant for decreased breath sounds on the right and a large, reducible ventral hernia.

Admission laboratories were largely unremarkable with renal function panel showing elevated blood glucose of 111 mg/dl. The Complete Blood Count showed normal white blood cell count of 6.84 K/ul , haemoglobin of 12.1 g/dl, and platelets of 492 K/ul. Respiratory Biofire for viral etiologies was negative. Troponins were unremarkable at 4 ng/l. Due to the history of recurrent pneumothorax, alpha 1-antitrypsin was measured and found to be within normal limits at 197 mg/dl.

**Figure 1:**
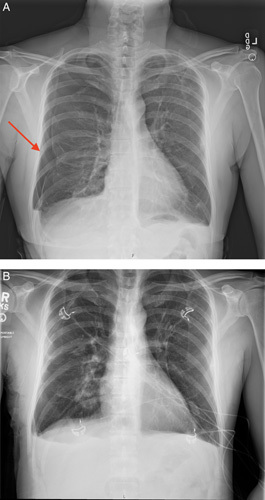
(A) Moderate sized right pneumothorax on presentation (left). (B) Resolution of right-sided pnseumothorax.

**Figure 2:**
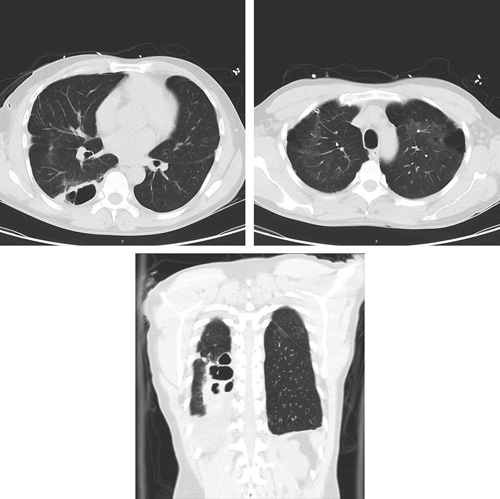
(High resolution CT Scan of chest) Diffuse emphysematous changes, bullous changes of the lateral aspect of both upper lobes, greater on the left, nonspecific mild ground glass infiltrates in the posterior aspect of the right lower lobe with adjacent bullous changes but resolution of pneumothorax. CT, computed tomography.

The admission chest X-ray (CXR) revealed a moderate right-sided pneumothorax without mediastinal shift [Figure 1, A]. A 12 French Pigtail chest tube was placed with immediate symptomatic relief by the pulmonary critical care fellow following the case. The follow-up CXR showed significant improvement of right-sided pneumothorax with only a tiny residual pneumothorax remaining. Cardiothoracic surgery was consulted in the emergency department, but found that the patient did not meet any criteria for cardiothoracic intervention at this time. A computed tomography (CT) scan of the chest demonstrated diffuse emphysematous and bullous changes of the lateral aspect of both upper lobes (greater on the left), nonspecific mild ground glass infiltrates in the posterior aspect of the right lower lobe with adjacent bullous changes, and resolution of the pneumothorax. Incidentally, a thick-walled cavitary lesion was also observed in the posterior right lower lung field [Figure 2]. After 48 h, the patient’s chest tube was placed to water seal, clamped, then removed without any significant issues. In addition, he was placed on non rebreather mask 100% oxygen to augment complete resolution for 12 h. His follow-up CXR did not show any evidence of pneumothorax. [Figure 1, b]. A barium swallow was performed to rule out possible aspiration or oesophageal aetiology for his thick-walled lesion. The barium swallow did not show any evidence of aspiration or oesophageal perforation.

The patient made a significant clinical recovery and was subsequently discharged on room air. A follow-up with the pulmonology department was planned with an interval scan and a discussion for consideration of lung pleurodesis, given his high risk of recurrence. Patient declined pleurodesis during this hospitalization. Patient felt at this time pleurodesis was unnecessary and future pneumothoraces could be prevented with adequate lifestyle modifications (i.e. marijuana smoking cessation). The patient was discharged with 4 weeks of amoxicillin/clavulanate potassium 500 mg treatment for potential infectious aetiology given his cavitary lesion. Separately, general surgery elected not to operate on ventral hernia because there were no signs of incarceration and the patient was spontaneously passing gas and having a bowel movement. The surgery plan was to have the patient follow-up outpatient at a hernia repair centre for an elective repair.

## Discussion

There is increasing evidence on the impact of marijuana on pulmonary physiology along with development of respiratory disorders. The common respiratory symptoms associated with marijuana use are likely due to physiological changes to the airway mucosa. Along with bronchodilation, histopathologic findings on long-term marijuana smokers demonstrate goblet cell hyperplasia, loss of ciliated epithelial cells, and intraepithelial/subepithelial inflammation. Metaplastic and nuclear changes were also found histopathologically^3^. Use of marijuana alone was also found to increase risk of outpatient visits for respiratory illnesses and increased incidence of acute bronchitic episodes^4^.

As for concomitant use of tobacco and marijuana, current research is contradictory. Some studies have shown that use of both marijuana and tobacco products increase the likelihood of chronic respiratory symptoms when compared with smokers of each substance separately^4^, but an attenuation of injury of marijuana on tobacco smokers has also been demonstrated. It is also interesting to note the effect heavy marijuana use may have on lung function in older patients. It was found that in men and women older than 40, heavy marijuana use was associated with a faster decline in Forced Expiratory Volume in 1 second when compared with never-smokers^5^. Ultimately, it can be concluded that tobacco may be the primary cause of lung injury, while marijuana has an unclear effect^4^. Although, in the case of our patient, tobacco use was minimal while marijuana use was high—greater than 7 g per day.

In regards to the incidence of pneumothoraces in habitual marijuana smokers, the mechanism may be attributed to the deep inhalation and prolonged breath holding typically practiced with marijuana inhalation and the formation of bullous lung disease^3–9^. Cases of large apical lung bullae have been associated with heavy marijuana smokers without significant tobacco use^6^. Another study reported an increase of pneumothorax in young marijuana smokers^3^. Chest radiographs of this population demonstrated large bullae that were not present in non-smokers^3^. Additionally, deep inhalation along with prolonged breath holding when smoking marijuana may lead to alveolar rupture. If the smoker also performs the Valsalva manoeuvre during inhalation, the risk of barotrauma increases^4^.

In our patient, other risk factors to consider are low BMI and previous history of a complicated abdominal hernia repair. Low BMI may be associated with a risk of primary spontaneous pneumothorax recurrence suggesting that BMI may be a helpful predictor of risk^12^. A CT-chest demonstrated bullous changes on the lateral aspect of both upper lung lobes with greater changes on the left along with diffuse emphysematous changes. Additionally, lack of an abdominal wall may cause diaphragmatic dysfunction. The abdominal wall assists the diaphragm on inspiration by increasing abdominal pressure when the diaphragm contracts. This dysfunction may present as dyspnoea or platypnea attributed to atelectasis or deconditioning^13^. Given our patient’s numerous risk factors, including low BMI and lack of abdominal wall strength, it is likely that concomitant marijuana and tobacco use led to the development of bullae and the recurrence of his spontaneous pneumothorax.

## Ethical approval

Ethical approval was permitted by the University Medical Center of Southern Nevada for conduction of this study.

## Consent

Authors received permission from the patient pertaining release of protected health information, photograph and video release prior to writing of the case study.

## Source of funding

No external or internal funding influenced the conduction of this study.

## Conflicts of interest disclosure

Authors have no conflicts of interests to disclose with this case report.

## Research registration unique identifying number (UIN)

Does not apply.

## Provenance and peer review

Not commissioned, externally peer-reviewed.

## Acknowledgements

The authors thank Kirk Kerkorian School of Medicine, Department of Internal Medicine.
